# Heterogeneity of Checkpoint Inhibitor–Associated Pneumonitis: A Multicenter Study on Inflammatory Subtypes and Clinical Outcomes

**DOI:** 10.1002/cam4.71041

**Published:** 2025-07-16

**Authors:** Tingyue Luo, Tiantian Liu, Weisheng Chen, Wei Lei, Shuquan Wei, Yan Wang, Shudong Ma, Li Lin, Junjie Xi, Zeyu Fu, Jingze Guo, Qiang Xiao, Xiaodan Chang, Danhui Huang, Shifang Yang, Laiyu Liu, Shaoxi Cai, Hangming Dong

**Affiliations:** ^1^ Chronic Airways Diseases Laboratory, Department of Respiratory and Critical Care Medicine Nanfang Hospital, Southern Medical University Guangzhou Guangdong China; ^2^ Department of General Surgery Nanfang Hospital, Southern Medical University Guangzhou Guangdong China; ^3^ Pulmonary and Critical Care Medicine Shunde Hospital, Southern Medical University (The First People's Hospital of Shunde Foshan) Foshan Guangdong China; ^4^ Department of Pulmonary and Critical Care Medicine Guangzhou First People's Hospital, School of Medicine, South China University of Technology Guangzhou Guangdong China; ^5^ Guangdong Second Provincial General Hospital Guangzhou Guangdong China; ^6^ Department of Oncology Nanfang Hospital, Southern Medical University Guangzhou Guangdong China; ^7^ Department of Pulmonary and Critical Care Medicine Guangdong Provincial People's Hospital, Southern Medical University Guangzhou Guangdong China

**Keywords:** blood inflammation subtypes, checkpoint inhibitor–associated pneumonitis, clinical characteristics, heterogeneity, outcomes

## Abstract

**Introduction:**

Checkpoint inhibitor–associated pneumonitis (CIP) is a serious manifestation following tumor immunotherapy, typically mediated by T lymphocytes. However, the heterogeneity in blood inflammatory profiles and its implications on clinical characteristics and outcomes for CIP remain underexplored.

**Methods:**

This multicenter retrospective study included 113 CIP patients without pathogen infection, aiming to classify CIP based on peripheral blood immune cell percentages.

**Results:**

We identified three inflammatory subtypes: normal type (42.5%), neutrophil type (41.6%), and eosinophil type (15.9%). The normal‐type pneumonitis primarily occurred in Grades 1–2 (79.2%), with nonspecific interstitial pneumonia radiological features, and demonstrated a significant recovery to normal lung status after effective treatment (*p* < 0.001). In contrast, patients with the neutrophil type were associated with symptomatic onset (*p* = 0.036), cryptogenic organizing pneumonitis on imaging (*p* = 0.004), higher steroid doses (> 40 mg/day, *p* = 0.011), anti‐fibrotic treatments (*p* = 0.007), and pneumonia‐related mortality rate was 27.7% (*p* = 0.037). Eosinophil‐type pneumonitis was more common in patients with chronic pulmonary inflammation, typically Grades 1–2 (66.7%) with hypersensitivity pneumonitis patterns (*p* < 0.001) and better prognosis.

**Conclusion:**

In summary, our study reveals that CIP can be classified into normal, neutrophil, and eosinophil types, each with distinct treatment responses and prognoses. These findings underscore the importance of subtype‐specific treatment strategies and highlight the prognostic significance of early intervention in both normal and eosinophil subtypes.

**Trial Registration:** ClinicalTrials.gov identifier: ChiCTR2200065859.

## 1 Introduction

Checkpoint inhibitor–associated pneumonitis (CIP) is one of the most serious adverse reactions following tumor immunotherapy [1, 2, 3]. With the widespread use of immune checkpoint inhibitors (ICIs) [4, 5], the incidence of CIP has increased annually to 13%–30% [6, 7, 8]. Owing to the resemblance of clinical symptoms, radiographic characteristics, and pathological features to those of other forms of pneumonitis, the diagnostic criteria and treatment strategies for CIP are not specific [1, 2, 3]. Consequently, CIP is the most prevalent fatal adverse event associated with ICI treatment [1, 9], with a mortality rate accounting for approximately one‐third of immune‐related adverse reactions (irAEs) [3, 10]. Therefore, further analysis and research on the characteristics of this disease are urgently needed.

Numerous clinical sample tests have provided substantial evidence that T lymphocytes play a central role in the development of CIP [11, 12, 13, 14]. However, recent studies have reported that in addition to T cells, other immune cells also contribute to the onset of CIP. For example, patients with CIP present a significant increase in the percentage of eosinophils in both peripheral blood and bronchoalveolar lavage fluid (BALF), and the regression of pneumonitis coincides with a decrease in eosinophils [15, 16, 17, 18]. In addition, neutrophils were also found to be elevated in CIP patients, and the imaging grades of pneumonitis were predominantly Grades 2–3 [12, 19, 20]. Moreover, several studies have demonstrated that a high ratio of neutrophils to lymphocytes in the peripheral blood is associated with greater severity and poorer prognosis of CIP [21, 22]. All of these studies indicate that there is inflammatory cell heterogeneity in the peripheral blood of CIP patients, which might be an important basis for CIP classification. Furthermore, different inflammatory subtypes may be related to different clinical characteristics and prognoses. However, currently, no reports have considered associations between peripheral blood inflammatory subtypes and CIP simultaneously. Therefore, we conducted a multicenter retrospective study to investigate the clinical characteristics and outcomes of CIP patients on the basis of their blood inflammatory subtypes.

## 2 Methods

### 2.1 Study Design

**FIGURE 1:**
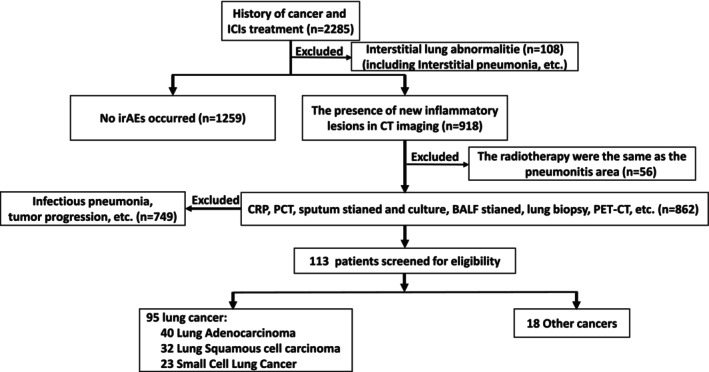
Study design and participating patients. Flowchart showing study enrollment and adjudication of patients into inclusion groups. ICIs, immune checkpoint inhibitors.

This study was conducted at five medical centers in China from January 1, 2020, to December 31, 2023. The deadline for follow‐up was March 31, 2024. The median follow‐up time was 649 days. The inclusion and exclusion criteria for patients with CIP were jointly formulated by radiologists, treating physicians, and laboratory physicians according to the guidelines [23, 24, 25], as described in Figure 1. Briefly, we enrolled patients with CIP who had no pathogen infections and whose immune cell percentages remained stable (≤ 5% variation) over at least 3 months before the occurrence of CIP. Patients with CIP were excluded if they had radiation pneumonitis, interstitial lung disease, other abnormalities, severe chronic obstructive pulmonary disease, connective tissue diseases, allergic conditions (atopic dermatitis, bronchial asthma, etc.), or autoimmune conditions. The inclusion of control group patients met the following criteria: (1) They were matched with the CIP group patients by sex and age; (2) they received the same duration of treatment with ICIs as the CIP group but never experienced any irAEs. Peripheral blood samples were collected within 24 h of CIP diagnosis and prior to the initiation of any immunosuppressive therapy (e.g., steroids) or chemotherapy, and the blood data from the control group corresponded to the same period of treatment. The treatment and management of CIP are based on guidelines [23] and consensus [24, 25]. In addition to steroid therapy, anti‐fibrotic drugs (including nintedanib or pirfenidone) and immunosuppressants (mycophenolate mofetil) could be considered for use when patients were steroid refractory [26, 27], steroid dependent [28], or had imaging features of pulmonary fibrosis.

We collected sex, age, history of lung disease, therapeutic regimen, and imaging data from all patients. The study protocol was approved by the Ethics Committee of Nanfang Hospital of Southern Medical University, and written informed consent was obtained from the patients and/or guardians in accordance with the Declaration of Helsinki before the study. This study was registered at www.chictr.org.cn as #ChiCTR2200065859.

### 2.2 Definitions

Inflammatory subtypes were defined by elevated percentages (> 5%) of immune cells in the peripheral blood when CIP occurs, which compared to the average percentage of three previous times (when no infection was present). To calculate the percentage of inflammation classification more accurately, we chose to include the control group and set the cutoff point for the percentage of peripheral blood cells as the 90th percentile of the control group. Finally, three inflammatory subtypes were defined: samples with a neutrophil percentage > 70.5% as the neutrophil type and those with an eosinophil percentage > 5.3% as the eosinophil type. The normal type was defined as the percentage of each immune cell within the normal range (< 5%). Additionally, some patients exhibited increased percentages of more than two types of immune cells, and their sputum smears frequently showed abnormal percentages of cocci and rods. Given the potential for local infection in these cases and the resulting complexity, these patients were excluded from the analysis of inflammation classification.

According to previous reports [10, 29, 30, 31, 32, 33], two radiologists with over 10 years of experience independently classified the imaging characteristics of CIP into four categories: cryptogenic organizing pneumonitis (COP), acute interstitial pneumonitis/acute respiratory distress syndrome (AIP/ARDS), hypersensitivity (HP), and nonspecific interstitial pneumonitis (NSIP). When a difference existed between the two radiologists' opinions, a final decision was made after consultation with the director of the department of radiology. We divided the imaging prognosis of pulmonary inflammation patients into three categories on the basis of clinical observations: recovery, chronic inflammation, and progression. Recovery was defined as the resolution of lung inflammation on imaging examinations from the onset of CIP until the end of follow‐up. Chronic inflammation was defined as the period from the onset of CIP to the end of follow‐up; the lung inflammation was controlled, and a local chronic inflammation area formed at the original inflammation site. Progression was defined as the situation in which lung inflammation had not been controlled after the onset of CIP, even with the use of high‐dose systemic corticosteroids. All radiologists involved in the evaluation of imaging features were unaware of the patients' inflammatory classification and treatment status.

### 2.3 Statistical Analysis

SPSS 26.0 software (SPSS, Chicago, IL) was used for the statistical analysis. Quantitative variables were expressed as medians (ranges) and qualitative variables were expressed as percentages. Pearson's Chi‐square test or Fisher's exact test was used for categorical data. Kaplan–Meier survival analysis was used to calculate cumulative survival, and the log‐rank test was used to compare the survival rates of two or more groups. Univariate and multivariate Cox regression analyses were used to identify factors associated with prognosis. In the univariate analysis, variables with *p* < 0.1 were considered significant and were subsequently incorporated into the multivariate model for further examination. The final results were reported as statistically significant when *p* < 0.05.

## 3 Results

### 3.1 Cohort Characteristics

A total of 113 CIP patients were enrolled from January 2020 to December 2023. There were no differences between the CIP group and the control group in terms of cancer type, PD‐L1 expression status, or therapeutic regimen. In terms of smoking habits, patients who had smoked appeared to be more likely to develop CIP (*p* = 0.017). In addition, patients with chronic pulmonary inflammation or pulmonary nodules may be more likely to develop CIP, whereas those without underlying lung diseases were less likely to develop CIP (*p* < 0.001) (Table S1). The median time from the first use of ICIs to the onset of CIP was 120 days (range, 6–577 days), and Grade 2 CIP was the most common (51.3%) (Figure S1A). With respect to the clinical symptoms experienced at the onset of CIP, cough was the most common symptom (51.3%), followed by expectoration (40.7%). Notably, 37.2% of the patients did not exhibit any obvious clinical symptoms when CIP occurred (Figure S1B). Further analysis revealed that 73.8% of these asymptomatic patients had Grades 1–2 pneumonitis (Figure S2). Among the ICIs used at the onset of CIP, pembrolizumab accounted for the highest percentage at 23.9%, followed by tirelizumab (23.0%), sintilimab (17.7%), camrelizumab (14.2%), and duvalizumab (6.2%) (Figure S1C). The overall survival rates of the CIP group and control group were shown in Figure S1D. The 2‐year cumulative survival rate of the CIP group was 54.7%, whereas the control group had a higher rate of 69.8%. In terms of radiographic classification of pneumonitis, the most common radiographic classification of pneumonitis was COP (31.9%), followed by AIP/ARDS (28.3%), NSIP (23.0%) and HP (16.8%) (Figure S1E).

### 3.2 Inflammation Subtypes of CIP

**FIGURE 2:**
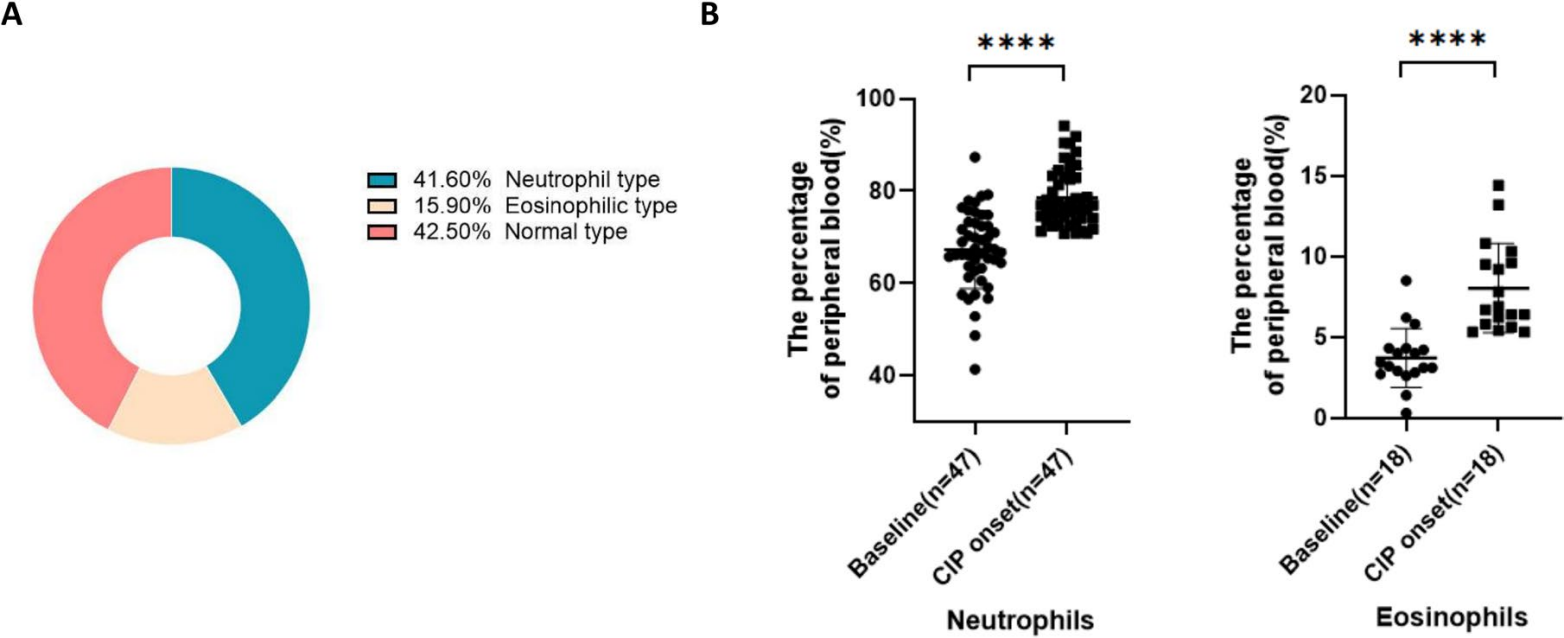
Inflammatory phenotype of CIP. (A) Inflammation classification based on the proportion of peripheral blood cells. (B) Dot plots show the percentages of immune cells in the inflammatory classification before and at the onset of CIP. CIP, checkpoint inhibitor pneumonitis.

Three inflammation subtypes were defined on the basis of the percentages of immune cells in the peripheral blood, using the 90th percentile values obtained from the control group (Figure 2A). The most common subtype was the normal type (42.5%), followed by the neutrophil type (41.6%) and the eosinophil type (15.9%). Each inflammatory subtype exhibited a significant increase in the corresponding immune cell population before and at the onset of CIP (Figure 2B).

### 3.3 Clinical Characteristics of Inflammatory Subtypes

Baseline characteristics varied among the different inflammatory subtypes (Table 1). Patients with chronic pulmonary inflammation were more likely to have the eosinophil type than those with a normal type (*p* = 0.024). Regarding smoking habits, primary cancer type, PD‐L1 expression, therapeutic regimens, and history of chest radiotherapy, no significant differences were found among the three inflammatory subtypes.

**TABLE 1:** Baseline characteristics in inflammatory subtypes of CIP.

Variables (*n* (%))	Neutrophil type	Eosinophil type	Normal type	*p*
No. subjects	47	18	48	
Gender				0.651
Male	43 (91.5)	15 (83.3)	43 (89.6)	
Female	4 (8.5)	3 (16.7)	5 (10.4)	
Age at CIP diagnosis				0.988
< 65	27 (57.4)	10 (55.6)	27 (56.2)	
≥ 65	20 (42.6)	8 (44.4)	21 (43.8)	
Smoking habits				0.284
Current/former	36 (76.6)	16 (88.9)	42 (87.5)	
Never	11 (23.4)	2 (11.1)	6 (12.5)	
History of chest radiotherapy				0.569
Yes	18 (38.3)	8 (44.4)	15 (31.3)	
No	29 (61.7)	10 (55.6)	33 (68.7)	
History of lung disease				
Chronic pulmonary inflammation	18 (38.3)	9 (50.0)^a^	9 (18.8)^b^	**0.024**
COPD	5 (10.6)	1 (5.6)	6 (12.5)	
Chronic inflammation	13 (27.7)	7 (38.8)	3 (6.3)	
Asthma	0	1 (5.6)	0	
Emphysema	10 (21.3)	4 (22.2)	14 (29.2)	
Other comorbidities^1^				0.895
Yes	40 (85.1)	15 (83.3)	42 (87.5)	
No	7 (14.9)	3 (16.7)	6 (12.5)	
Primary cancer type				0.147
Lung cancer	36 (76.6)	17 (94.4)	42 (87.5)	
Adenocarcinoma	16 (34.0)	7 (38.9)	17 (35.4)	
Squamous	12 (25.6)	7 (38.9)	13 (27.1)	
Small cell carcinoma	8 (17.0)	3 (16.6)	12 (25.0)	
Others^2^	11 (23.4)	1 (5.6)	6 (12.5)	
Cancer stage				0.086
II	1 (2.2)	0 (0)	2 (4.2)	
III	27 (57.4)	14 (77.8)	20 (41.7)	
IV	19 (40.4)	4 (22.2)	26 (54.1)	
PD‐L1 expression status				0.209
Positive (TPS ≥ 1%)	15 (31.9)	9 (50.0)	19 (39.6)	
Negative (TPS < 1%)	12 (25.5)	2 (11.1)	7 (14.6)	
Therapeutic regimen				0.089
ICIs combination therapy^3^	38 (85)	12 (66.7)	43 (89.6)	
ICIs monotherapy	9 (15)	6 (33.3)	5 (10.4)	

*Note:* a, b: a statistical difference between the two groups. To make the statistically significant results more prominent.

Abbreviations: COPD, chronic obstructive pulmonary disease; ICIs, immune checkpoint inhibitors; PD‐L, programmed cell death ligand.

^1^ Other comorbidities included hypertension, diabetes, anemia, nodules, etc.

^2^ Others included gastric cancer, esophageal cancer, nasopharyngeal cancer, etc.

^3^ Combination therapy including cisplatin + pemetrexed or carboplatin + paclitaxel, etc.

Furthermore, different inflammatory subtypes presented distinct clinical characteristics (Table 2 and Figure S3). In summary, at the onset of CIP, the neutrophil type was significantly associated with clinical symptoms, such as cough, expectoration, or shortness of breath (*p* = 0.036). For imaging grades, eosinophils and normal types were mostly Grades 1–2 numerically. Notably, according to the radiologic classification (*p* < 0.001), the neutrophil type was observed more frequently in cases of COP (*p* = 0.004), and the eosinophil type was predominantly associated with HP (*p* < 0.001), while the normal type was mostly found in NSIP numerically. Additionally, regarding the induction of different inflammatory subtypes by ICIs, the neutrophil type was most frequently observed with pembrolizumab and tirelizumab (27.7% and 25.5%, respectively), the eosinophil type was more commonly associated with tirelizumab (37.5%), and the normal type did not seem to be related to certain ICIs (Figure S4A). However, it should be noted that these differences were not statistically significant. Analysis of the main inflammatory subtypes induced by each ICI showed that camrelizumab primarily induced the neutrophil type and normal type (46.7% and 46.7%, respectively); pembrolizumab mainly induced the neutrophil type (48.1%), whereas sintilimab mainly induced the normal type (60.0%). Tirelizumab was also found to induce mainly the neutrophil type and normal type (48.0% and 40.0%, respectively) (Figure S4B).

**TABLE 2:** Clinical features in inflammatory subtypes of CIP.

Clinical characteristics (*n* (%))	Neutrophil type	Eosinophil type	Normal type	*p*
No. subjects	**47**	**18**	**48**	
Symptoms				**0.036**
Symptomatic	36 (76.6)^a^	9 (50.0)^b^	26 (54.2)^b^	
Asymptomatic	11 (23.4)^a^	9 (50.0)^b^	22 (45.8)^b^	
Imaging grades				0.075
Grades 1–2	27 (57.4)	12 (66.7)	38 (79.2)	
Grades 3–4	20 (42.6)	6 (33.3)	10 (20.8)	
Radiologic classification				**< 0.001**
COP	22 (46.8)^a^	1 (5.6)^b^	13 (27.1)	
AIP/ARDS	15 (31.9)	4 (22.2)	13 (27.1)	
HP	2 (4.3)^a^	9 (50.0)^b^	8 (16.7)^a^	
NSIP	8 (17.0)	4 (22.2)	14 (29.2)	
Immune checkpoint inhibitors				0.375
Camrelizumab	7 (14.9)	1 (5.6)	7 (14.6)	
Pembrolizumab	13 (27.7)	5 (27.8)	9 (18.8)	
Sintilimab	4 (8.6)	4 (22.2)	12 (25.0)	
Tirelizumab	12 (25.5)	3 (37.5)	10 (20.8)	
Steroid‐effective dose				**0.011**
≤ 40 mg/day	14 (29.8)^a^	9 (50.0)	29 (60.4)^b^	
> 40 mg/day	33 (70.2)^a^	9 (50.0)	19 (39.6)^b^	
Anti‐fibrotic drugs				**0.007**
Used	14 (29.8)^a^	2 (11.1)	3 (6.3)^b^	
Unused	33 (70.2)^a^	16 (88.9)	45 (93.7)^b^	
Imaging prognosis				**< 0.001**
Recovery	10 (21.3)^a^	8 (44.4)	33 (68.8)^b^	
Chronic inflammation	16 (34.0)	5 (27.8)	9 (18.8)	
Progressive	21 (44.7)^a^	5 (27.8)	6 (12.5)^b^	
Deaths related to pneumonia				**0.037**
Yes	13 (27.7)^a^	5 (27.8)	4 (8.3)^b^	
No	34 (72.3)^a^	13 (72.2)	44 (91.7)^b^	

*Note:* a, b: a statistical difference between the two groups. To make the statistically significant results more prominent.

Abbreviations: AIP/ARDS, acute interstitial pneumonia/acute respiratory distress syndrome; COP, cryptogenic organizing pneumonia‐like; HP, hypersensitivity; NSIP, nonspecific interstitial pneumonia.

### 3.4 Treatment and Outcomes of Inflammatory Subtypes

**FIGURE 3:**
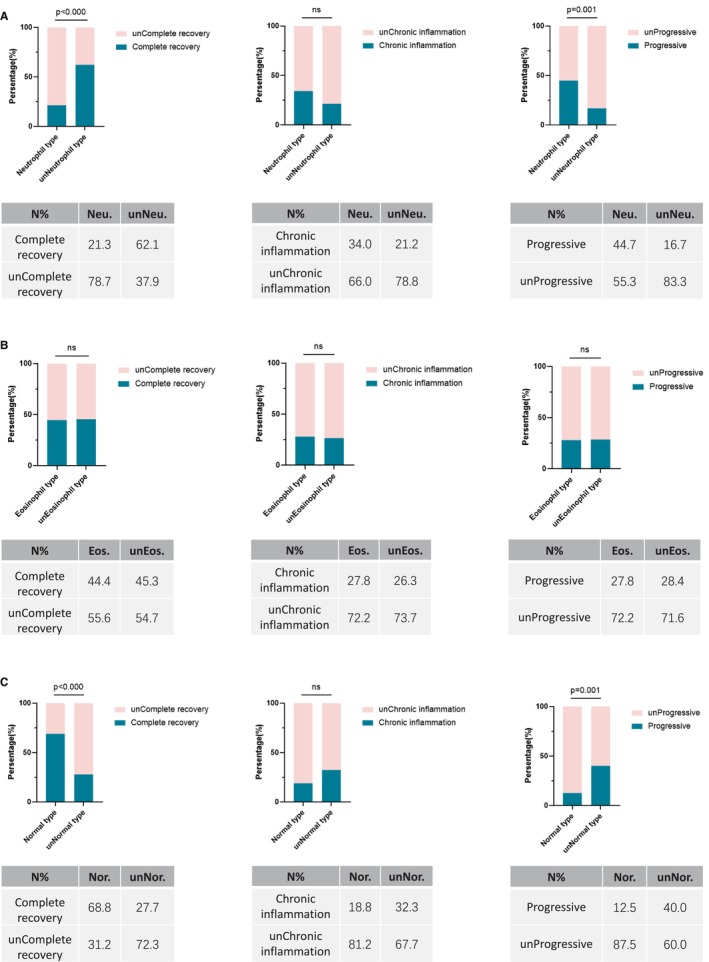
Imaging prognosis of each inflammatory subtype. From top to bottom were: neutrophil type, eosinophil type, and normal type. unNeutrophil refers to the non‐neutrophil type (including eosinophil type and normal type). unEosinophil refers to the non‐eosinophil type. unNormal refers to the non‐normal type. unCompleted refers to not complete. unChronic refers to not chronic. unProgressive refers to not progressive.

There were also differences in treatment options and outcomes among the subtypes (Table 2). In terms of the effective dose of steroids for the treatment of CIP, 60.4% of the normal type patients were able to effectively control inflammation with an initial dose of ≤ 40 mg/day. However, 70.2% of the patients with the neutrophil type required a higher dose (> 40 mg/day) of steroids to control the disease (*p* = 0.011). Notably, about one‐third of the neutrophil type required anti‐fibrotic drugs to control lung inflammation progression, which was significantly less common in the normal type (*p* = 0.007). Moreover, the imaging prognosis of each subtype was different (*p* < 0.001). The neutrophil type had the lowest complete recovery rate at 21.3% (*p* < 0.001) and the highest progression rate at 44.7% (*p* = 0.001) (Figure 3) and was often followed by chronic inflammation or fibrosis after inflammation control. Interestingly, among patients with the normal type, the complete recovery rate of lung imaging was significantly higher at approximately 68.8% (*p* < 0.001) (Figure 3).

Notably, there was a significant difference in the number of deaths associated with CIP (*p* = 0.037) (Table 2). Among them, there were intergroup differences between the neutrophil type and normal type, and the neutrophil type of CIP had a higher mortality rate. Additionally, univariate and multivariate Cox regression analyses revealed that inflammatory subtype (*p* = 0.042), cancer stage (*p* = 0.008), and abnormal CRP (C‐reactive protein) level (*p* = 0.036) were risk factors affecting prognosis (Table 3).

**TABLE 3:** Cox regression analysis of the association between demographic characteristics and CIP subtype with death related to pneumonia.

Variables (*n* (%))	Univariate		Multivariable	
	HR (95% CI)	*p*	HR (95% CI)	*p*
Inflammatory subtypes^a^		**0.062**		**0.042**
Eosinophil type	1.112 (0.393–3.144)	0.842	0.668 (0.226–1.977)	0.466
Normal type	0.294 (0.078–1.103)	0.070	0.193 (0.050–0.745)	0.017
Primary cancer type	4.715 (0.627–35.427)	0.132		
Cancer stage^b^		**0.082**		**0.008**
II	0	0.985	0	0.985
III	0.360 (0.147–0.881)	0.025	0.227 (0.089–0.577)	0.002
History of lung disease^c^	0.692 (0.270–1.775)	0.444		
Other comorbidities	25.503 (0.095–6832.600)	0.256		
History of chest radiotherapy	1.285 (0.551–2.997)	0.562		
CIP grade^d^	1.754 (0.757–4.064)	0.190		
CRP	3.440 (1.009–11.728)	**0.048**	3.977 (1.087–14.544)	**0.036**

*Note:* To make the statistically significant results more prominent.

^a^ For inflammatory subtypes, the neutrophil type was used as the reference point for calculating.

^b^ For cancer stage, IV was used as the reference point for calculating.

^c^ For history of lung disease, classify based on the presence or absence of obstructive pneumonia.

^d^ For CIP grade, a two‐tier classification was used for statistical calculations (Grades 1–2 and Grades 3–4).

## 4 Discussion

This study was the first to classify CIP based on peripheral blood inflammation types and summarize the clinical characteristics of each subtype, focusing particularly on their impact on treatment response, imaging prognosis, and survival (Table 4). This approach provides a valuable theoretical foundation for the development of precise treatments for CIP, an area previously unexplored in epidemiology. We identified three distinct inflammatory subtypes of CIP, each with unique clinical features that significantly influence treatment outcomes, the severity and classification of pneumonitis on imaging, and CIP‐related survival rates. These findings highlight the importance of identifying inflammatory subtypes to tailor treatments, assess efficacy, and improve prognosis prediction.

**TABLE 4:** The inflammatory phenotype of checkpoint inhibitor–associated pneumonitis.

	History of lung disease	Clinical symptoms	Radiologic subtypes	Steroid effective dose	Anti‐fibrotic drugs	Imaging remission
Neutrophil type	—	Usual	COP	> 40 mg/day	Need	Progressive
Eosinophil type	Chronic pulmonary inflammation	—	HP	—	—	—
Normal type	—	—	—	≤ 40 mg/day	Rarely	Recovery

Abbreviations: COP, cryptogenic organizing pneumonia‐like; HP, hypersensitivity.

While numerous studies have reported that CIP was caused by hyperactive T lymphocytes [14, 34, 35, 36], interestingly, our study revealed that increased lymphocytes in peripheral blood were not common at the onset of CIP. This finding aligns with the results reported by Wang et al. [37], who compared irAEs with those of healthy individuals and reported no significant difference in lymphocyte counts in the peripheral blood. The potential explanation for this discrepancy may be due to lymphocytes being drawn to and accumulating in the lungs, thus reducing their proportion in the peripheral circulation. This hypothesis is supported by Kowalski et al., who found that CIP patients had decreased lymphocyte percentages in peripheral blood but increased levels in BALF [19, 38]. Further research is needed to elucidate the mechanisms behind this phenomenon and its implications for treatment response.

For CIP patients with elevated eosinophils, a case report was first published by Hara et al. in 2018, which reported that one CIP patient had an elevated percentage of eosinophils in both the peripheral blood and BALF [15]. In recent years, Jodai et al. and Chu et al. reported that there was an elevated percentage of eosinophils in the peripheral blood of CIP patients and that these patients could regress pneumonitis after steroid therapy [14, 15, 16, 17]. Similarly, in this research, eosinophils increased in 15.9% of patients at the onset of CIP, and these patients were generally sensitive to steroids. Interestingly, the regression of pneumonitis and reduction in eosinophils occurred simultaneously after steroid therapy, and the prognosis was best. These findings suggest that eosinophil type CIP patients may benefit from lower doses of steroids for shorter durations, highlighting the potential for personalized treatment strategies based on inflammatory subtypes. Additionally, the higher incidence of HP in eosinophil type CIP might be due to the activation of type 2 inflammation caused by ICIs, which lead to elevated and activated eosinophils, presenting with radiological features of allergic pneumonia. Recently, the Fleischner Society [39] proposed that the eosinophilic pneumonia pattern was a distinct imaging phenotype in drug‐related interstitial pneumonia. In our study, the eosinophil subtype pneumonia might have similar imaging features. However, referring to the classification from CIP‐specific literature, the currently recognized types are four: COP, AIP/ARDS, HP, and NSIP. To ensure clinical relevance, we did not include the eosinophilic pneumonia pattern in the imaging classification. Notably, none of our eosinophil‐type CIP cases exhibited isolated eosinophilic pneumonia patterns, likely due to the multifactorial etiology of eosinophilic lung infiltrates.

Moreover, this study revealed that 41.6% of CIP patients had elevated neutrophils, which was consistent with the results of Kowalski et al. [19], who reported increased neutrophils in both the peripheral blood and BALF when CIP occurred. More importantly, Li et al. [20]. found that elevated peripheral blood neutrophils were a risk factor for CIP refractory to steroids. Consistent with this, in our study, the neutrophil type required higher doses of steroids and struggled to control lung inflammation within a short timeframe. These findings suggested that additional immunosuppressants beyond steroids may be more effective in treating neutrophilic CIP. Our study also found that patients with the neutrophil subtype need more anti‐fibrotic treatment. This might be because they were less sensitive to steroid therapy, similar to steroid‐unresponsive neutrophilic asthma patients. Their lung inflammation might not be well controlled by conventional steroids, leading to ongoing and worsening inflammation and thus requiring anti‐fibrotic drugs. This suggests that the neutrophil subtype in CIP treatment is a more challenging state needing further attention and research. Although most international guidelines did not recommend anti‐fibrotic drugs as a standard treatment for CIP, they could be considered if there was obvious fibrotic progression [24]. In this study, if patients did not respond well to traditional immunosuppressive treatment and were at risk of persistent inflammation and fibrosis, anti‐fibrotic drugs would be used for treatment, which helped to slow down the fibrotic process. Furthermore, we also concluded that neutrophil‐type CIP was usually accompanied by clinical symptoms, was more likely to be classified as COP on imaging, and had a poorer prognosis. These results lay the groundwork for further mechanistic studies to understand the role of neutrophils in CIP pathogenesis and to develop targeted therapies. Notably, elevated peripheral blood neutrophils are usually considered pathogenic infections, but the patients in our study had already been confirmed to have no respiratory pathogen infection at the onset of CIP, leaving the reason for the increase in neutrophils unclear [1, 40]. Therefore, further investigations are needed to explore additional potential mechanisms through subsequent sequencing of clinical samples and basic research.

For patients with increased percentages of more than two types of immune cells of CIP, they might have concurrent lung infections, so we excluded these patients from our study. In clinical work, we reported that in CIP patients with increased neutrophilia and coinfection, the severity of pneumonitis was concentrated in Grades 3–4, and high‐dose corticosteroids and antibiotics were needed to control inflammation [41]. Similarly, Guezour et al. [42] reported that patients with Grades 3–4 adverse reactions often develop uncontrollable pulmonary infections [38, 43, 44]. This might be attributed to impaired local defense mechanisms caused by inflammation and tissue damage, which increases susceptibility to infections and complicates treatment [45]. This finding suggests that for CIPs with elevated neutrophil counts, we must pay more attention, and incorporating additional anti‐fibrosis drugs in the early stages to manage inflammation and minimize damage might be beneficial [24, 25, 46]. Otherwise, the vicious cycle of lung barrier damage and pathogen infection severely affects patient survival. In addition, for normal‐type CIP patients, we recommend the use of steroids for treatment according to the guidelines for immune‐related adverse reactions.

The limitation of this study was that the patients received steroid therapy immediately after CIP diagnosis, which limited the ability to assess the long‐term reproducibility of the inflammatory phenotype in peripheral blood. Future prospective studies with follow‐up are needed to explain the changes in peripheral blood and lung imaging in patients treated with ICIs. Secondly, our study did not directly integrate blood subtypes with imaging classifications (e.g., dual‐classification models) or CIP grade, nor did it compare their predictive power for CIP‐related mortality. Future studies with larger sample sizes should further validate the comparison between blood subtypes and imaging patterns or CIP grade.

In summary, this study highlights the heterogeneity of inflammation in the peripheral blood of patients with CIP and the distinct clinical features exhibited by different subtypes. Inflammatory subtypes can be recognized on the basis of elevated percentages of immune cells in peripheral blood, which may reflect less lung inflammation than induced sputum or BALF does but is more easily accessible in clinical practice. Neutrophil type is associated with poorer prognosis and radiologically characterized by COP. Eosinophil type is linked to a more favorable outcome with like HP in imaging. The recognition and characterization of inflammatory subtypes in CIP have significant implications for clinical diagnosis and treatment research. Further mechanistic studies are warranted to explore the potential utility of peripheral blood analysis in assessing the inflammatory status and guiding treatment decisions in CIP patients.

## Author Contributions

T.L., T.L., W.C., and W.L. were responsible for the conception and design of the study, interpretation of the data from all medical centers, and wrote the main manuscript text. S.W., Y.W., S.M., L.L., Q.X., and S.Y. were accountable for all aspects of the work in each medical center, including contributing to the ethical review, and collection, analysis, and interpretation of the data, and was responsible for drafting significant portions of the figures. J.X., Z.F., J.G., X.C., and D.H. conducted the data analysis, which included data collection and processing, performed the statistical analysis and interpretation of the data, and drafted significant portions of the tables. H.D., S.C., and L.L. were responsible for the study supervision and were involved in the design of the study, the analysis of the data, and the writing of the introduction and discussion section. All the authors read and approved the final manuscript.

## Ethics Statement

The study was approved by the relevant institutional review boards (NFEC‐2022‐420), and written informed consent was obtained from each subject.

## Consent

All images or other personal or clinical details of the participants presented in this paper provided consent for publication by signing the informed consent form. Specific personal information has been hidden in the paper.

## Conflicts of Interest

The authors declare no conflicts of interest.

## Supporting information


**Figure S1.** Clinical features of patients with entire CIP group. (A) Time from first dose of ICIs therapy to the date of each grade CIP developed. (B) Clinical symptoms of CIP. (C) The ICIs of CIP. (D) The survival probability of CIP group and control group. (E) The radiographic classification of CIP. (F) The survival probability of four radiological features. ICIs, immune checkpoint inhibitors; CIP, checkpoint inhibitor pneumonitis.
**Figure S2.** Imaging grades of asymptomatic patients at the onset of CIP. 26.2% patients were in Grade 1, 47.6% in Grade 2, 19.1% in Grade 3 and 7.1% in Grade 4. CIP, checkpoint inhibitor pneumonitis.
**Figure S3.** Radiologic classification of each inflammatory subtype. (A) Neutrophil type; (B) eosinophil type; (C) normal type.
**Figure S4.** Immune checkpoint inhibitors of CIP. (A) Inflammatory subtypes could be induced by different ICIs; (B) Different ICIs could induce different inflammatory subtypes. ICIs, immune checkpoint inhibitors.
**Figure S5.** The survival probability of Inflammatory subtypes. (A) Survival curves of each inflammatory subtype. (B) Comparison of survival curves between neutrophil type and control group.

**Table S1.** Baseline characteristics of the study population.

## Data Availability

The data that support the findings of this study are available on request from the corresponding author. The data are not publicly available due to privacy or ethical restrictions.

## References

[1] M. A. Postow , R. Sidlow , and M. D. Hellmann , “Immune‐Related Adverse Events Associated With Immune Checkpoint Blockade,” New England Journal of Medicine 378 (2018): 158–168. 29320654 10.1056/NEJMra1703481

[2] S. Rashdan , J. D. Minna , and D. E. Gerber , “Diagnosis and Management of Pulmonary Toxicity Associated With Cancer Immunotherapy,” Lancet Respiratory Medicine 6 (2018): 472–478. 29856320 10.1016/S2213-2600(18)30172-3 PMC7341891

[3] D. Y. Wang , J. E. Salem , J. V. Cohen , et al., “Fatal Toxic Effects Associated With Immune Checkpoint Inhibitors: A Systematic Review and Meta‐Analysis,” JAMA Oncology 4 (2018): 1721–1728. 30242316 10.1001/jamaoncol.2018.3923 PMC6440712

[4] P. Gotwals , S. Cameron , D. Cipolletta , et al., “Prospects for Combining Targeted and Conventional Cancer Therapy With Immunotherapy,” Nature Reviews. Cancer 17 (2017): 286–301. 28338065 10.1038/nrc.2017.17

[5] K. Suresh , J. Naidoo , C. T. Lin , and S. Danoff , “Immune Checkpoint Immunotherapy for Non‐Small Cell Lung Cancer: Benefits and Pulmonary Toxicities,” Chest 154 (2018): 1416–1423. 30189190 10.1016/j.chest.2018.08.1048 PMC6335259

[6] M. C. Garassino , S. Gadgeel , E. Esteban , et al., “Patient‐Reported Outcomes Following Pembrolizumab or Placebo Plus Pemetrexed and Platinum in Patients With Previously Untreated, Metastatic, Non‐Squamous Non‐Small‐Cell Lung Cancer (KEYNOTE‐189): A Multicentre, Double‐Blind, Randomised, Placebo‐Controlled, Phase 3 Trial,” Lancet Oncology 21 (2020): 387–397. 32035514 10.1016/S1470-2045(19)30801-0

[7] N. A. Rizvi , M. D. Hellmann , J. R. Brahmer , et al., “Nivolumab in Combination With Platinum‐Based Doublet Chemotherapy for First‐Line Treatment of Advanced Non‐Small‐Cell Lung Cancer,” Journal of Clinical Oncology 34 (2016): 2969–2979. 27354481 10.1200/JCO.2016.66.9861 PMC5569693

[8] R. S. Herbst , G. Giaccone , F. de Marinis , et al., “Atezolizumab for First‐Line Treatment of PD‐L1‐Selected Patients With NSCLC,” New England Journal of Medicine 383 (2020): 1328–1339. 32997907 10.1056/NEJMoa1917346

[9] W. T. Atchley , C. Alvarez , S. Saxena‐Beem , et al., “Immune Checkpoint Inhibitor‐Related Pneumonitis in Lung Cancer: Real‐World Incidence, Risk Factors, and Management Practices Across Six Health Care Centers in North Carolina,” Chest 160 (2021): 731–742. 33621599 10.1016/j.chest.2021.02.032 PMC8411447

[10] M. Nishino , N. H. Ramaiya , M. M. Awad , et al., “PD‐1 Inhibitor‐Related Pneumonitis in Advanced Cancer Patients: Radiographic Patterns and Clinical Course,” Clinical Cancer Research 22 (2016): 6051–6060. 27535979 10.1158/1078-0432.CCR-16-1320 PMC5161686

[11] K. Suzuki , T. Yanagihara , K. Matsumoto , et al., “Immune‐Checkpoint Profiles for T Cells in Bronchoalveolar Lavage Fluid of Patients With Immune‐Checkpoint Inhibitor‐Related Interstitial Lung Disease,” International Immunology 32 (2020): 547–557. 32253426 10.1093/intimm/dxaa022

[12] K. Suresh , J. Naidoo , Q. Zhong , et al., “The Alveolar Immune Cell Landscape Is Dysregulated in Checkpoint Inhibitor Pneumonitis,” Journal of Clinical Investigation 129 (2019): 4305–4315. 31310589 10.1172/JCI128654 PMC6763233

[13] S. T. Kim , A. Sheshadri , V. Shannon , et al., “Distinct Immunophenotypes of T Cells in Bronchoalveolar Lavage Fluid From Leukemia Patients With Immune Checkpoint Inhibitors‐Related Pulmonary Complications,” Frontiers in Immunology 11 (2020): 590494. 33552049 10.3389/fimmu.2020.590494 PMC7859512

[14] R. Bai , N. Chen , X. Chen , et al., “Analysis of Characteristics and Predictive Factors of Immune Checkpoint Inhibitor‐Related Adverse Events,” Cancer Biology & Medicine 18 (2021): 1118–1133. 34259422 10.20892/j.issn.2095-3941.2021.0052 PMC8610160

[15] K. Hara , K. Yamasaki , M. Tahara , et al., “Immune Checkpoint Inhibitors‐Induced Eosinophilic Pneumonia: A Case Report,” Thoracic Cancer 12 (2021): 720–724. 33476070 10.1111/1759-7714.13848 PMC7919115

[16] T. Jodai , C. Yoshida , R. Sato , et al., “A Potential Mechanism of the Onset of Acute Eosinophilic Pneumonia Triggered by an Anti‐PD‐1 Immune Checkpoint Antibody in a Lung Cancer Patient,” Immunity, Inflammation and Disease 7 (2019): 3–6. 30461210 10.1002/iid3.238 PMC6416763

[17] X. Chu , J. Zhao , J. Zhou , et al., “Association of Baseline Peripheral‐Blood Eosinophil Count With Immune Checkpoint Inhibitor‐Related Pneumonitis and Clinical Outcomes in Patients With Non‐Small Cell Lung Cancer Receiving Immune Checkpoint Inhibitors,” Lung Cancer 150 (2020): 76–82. 33080551 10.1016/j.lungcan.2020.08.015

[18] X. Jia , Y. Zhang , T. Liang , et al., “Comprehensive Nomogram Models for Predicting Checkpoint Inhibitor Pneumonitis,” American Journal of Cancer Research 13 (2023): 2681–2701. 37424813 PMC10326584

[19] B. Kowalski , A. Valaperti , P. Bezel , et al., “Analysis of Cytokines in Serum and Bronchoalveolar Lavage Fluid in Patients With Immune‐Checkpoint Inhibitor‐Associated Pneumonitis: A Cross‐Sectional Case‐Control Study,” Journal of Cancer Research and Clinical Oncology 148 (2022): 1711–1720. 34347128 10.1007/s00432-021-03750-z PMC9189083

[20] Y. Li , X. Jia , Y. Zhang , et al., “Risk Factors and Immunomodulators Use in Steroid‐Refractory Checkpoint Inhibitor Pneumonitis,” Journal for Immunotherapy of Cancer 11 (2023): 11. 10.1136/jitc-2023-006982 PMC10254972 37290926

[21] L. Peng , Y. Wang , F. Liu , et al., “Peripheral Blood Markers Predictive of Outcome and Immune‐Related Adverse Events in Advanced Non‐Small Cell Lung Cancer Treated With PD‐1 Inhibitors,” Cancer Immunology, Immunotherapy 69 (2020): 1813–1822. 32350592 10.1007/s00262-020-02585-w PMC7413896

[22] X. Lin , H. Deng , Y. Yang , et al., “Peripheral Blood Biomarkers for Early Diagnosis, Severity, and Prognosis of Checkpoint Inhibitor‐Related Pneumonitis in Patients With Lung Cancer,” Frontiers in Oncology 11 (2021): 698832. 34327140 10.3389/fonc.2021.698832 PMC8313853

[23] J. A. Thompson , B. J. Schneider , J. Brahmer , et al., “Management of Immunotherapy‐Related Toxicities, Version 1.2022, NCCN Clinical Practice Guidelines in Oncology,” Journal of the National Comprehensive Cancer Network 20 (2022): 387–405. 35390769 10.6004/jnccn.2022.0020

[24] W. Wang , Q. Wang , C. Xu , et al., “Chinese Expert Consensus on the Multidisciplinary Management of Pneumonitis Associated With Immune Checkpoint Inhibitor,” Thoracic Cancer 13 (2022): 3420–3430. 36268845 10.1111/1759-7714.14693 PMC9715776

[25] Anticancer Drug‐induced Interstitial Lung Disease Management G , “Expert Consensus on the Diagnosis and Treatment of Anticancer Drug‐Induced Interstitial Lung Disease,” Zhonghua Zhong Liu Za Zhi 44 (2022): 693–702. 35880334 10.3760/cma.j.cn112152-20220412-00244

[26] J. Luo , J. A. Beattie , P. Fuentes , et al., “Beyond Steroids: Immunosuppressants in Steroid‐Refractory or Resistant Immune‐Related Adverse Events,” Journal of Thoracic Oncology 16 (2021): 1759–1764. 34265432 10.1016/j.jtho.2021.06.024 PMC8464489

[27] A. Balaji , M. Hsu , C. T. Lin , et al., “Steroid‐Refractory PD‐(L)1 Pneumonitis: Incidence, Clinical Features, Treatment, and Outcomes,” Journal for Immunotherapy of Cancer 9 (2021): e001731. 33414264 10.1136/jitc-2020-001731 PMC7797270

[28] J. Beattie , H. Rizvi , P. Fuentes , et al., “Success and Failure of Additional Immune Modulators in Steroid‐Refractory/Resistant Pneumonitis Related to Immune Checkpoint Blockade,” Journal for Immunotherapy of Cancer 9 (2021): e001884. 33568350 10.1136/jitc-2020-001884 PMC7878154

[29] J. Naidoo , X. Wang , K. M. Woo , et al., “Pneumonitis in Patients Treated With Anti–Programmed Death‐1/Programmed Death Ligand 1 Therapy,” Journal of Clinical Oncology 35 (2017): 709–717. 27646942 10.1200/JCO.2016.68.2005 PMC5559901

[30] J. Naidoo , M. Nishino , S. P. Patel , et al., “Immune‐Related Pneumonitis After Chemoradiotherapy and Subsequent Immune Checkpoint Blockade in Unresectable Stage III Non‐Small‐Cell Lung Cancer,” Clinical Lung Cancer 21 (2020): E435–E444. 32576443 10.1016/j.cllc.2020.02.025

[31] P. F. Cui , Z. F. Liu , G. Q. Wang , et al., “Risk Factors for Pneumonitis in Patients Treated With Anti‐Programmed Death‐1 Therapy: A Case‐Control Study,” Cancer Medicine 7 (2018): 4115–4120. 29797416 10.1002/cam4.1579 PMC6089164

[32] M. Nishino , H. Hatabu , F. S. Hodi , and N. H. Ramaiya , “Drug‐Related Pneumonitis in the Era of Precision Cancer Therapy,” JCO Precision Oncology 1 (2017): 1–12. 10.1200/PO.17.00026 PMC7446404 32913972

[33] M. Nishino , H. Hatabu , L. M. Sholl , and N. H. Ramaiya , “Thoracic Complications of Precision Cancer Therapies: A Practical Guide for Radiologists in the New Era of Cancer Care,” Radiographics 37 (2017): 1371–1387. 28898185 10.1148/rg.2017170015 PMC5621730

[34] C. Sung , J. An , S. Lee , et al., “Integrative Analysis of Risk Factors for Immune‐Related Adverse Events of Checkpoint Blockade Therapy in Cancer,” Nature Cancer 4 (2023): 844–859. 37308678 10.1038/s43018-023-00572-5

[35] D. B. Johnson and J. M. Balko , “T Cell Dynamism and Immune‐Related Adverse Events,” Cancer Cell 41 (2023): 658–659. 36868223 10.1016/j.ccell.2023.02.006

[36] F. Berner , D. Bomze , C. Lichtensteiger , et al., “Autoreactive Napsin A‐Specific T Cells Are Enriched in Lung Tumors and Inflammatory Lung Lesions During Immune Checkpoint Blockade,” Science Immunology 7 (2022): eabn9644. 36054337 10.1126/sciimmunol.abn9644

[37] H. Wang , F. Zhou , C. Zhao , et al., “Interleukin‐10 Is a Promising Marker for Immune‐Related Adverse Events in Patients With Non‐Small Cell Lung Cancer Receiving Immunotherapy,” Frontiers in Immunology 13 (2022): 840313. 35222434 10.3389/fimmu.2022.840313 PMC8863608

[38] Y. Feng , C. Chen , L. Zhao , X. Zhu , X. Zhu , and Q. Li , “A Potential Mechanism of the Onset of Immune‐Related Pneumonitis Triggered by Anti‐PD‐1 Treatment in a Patient With Advanced Adenocarcinoma Lung Cancer: Case Report,” BMC Pulmonary Medicine 21 (2021): 291. 34521373 10.1186/s12890-021-01649-6 PMC8438889

[39] T. Johkoh , K. S. Lee , M. Nishino , et al., “Chest CT Diagnosis and Clinical Management of Drug‐Related Pneumonitis in Patients Receiving Molecular Targeting Agents and Immune Checkpoint Inhibitors,” Chest 159 (2021): 1107–1125. 33450293 10.1016/j.chest.2020.11.027

[40] M. Perol , “Multidisciplinary Approach of Immune Checkpoint Inhibitor‐Related Pneumonitis: A Key to Address Knowledge and Management Gaps,” Journal of Thoracic Oncology 15 (2020): 1261–1264. 32718532 10.1016/j.jtho.2020.05.007 PMC7380946

[41] M. Delaunay , J. Cadranel , A. Lusque , et al., “Immune‐Checkpoint Inhibitors Associated With Interstitial Lung Disease in Cancer Patients,” European Respiratory Journal 50 (2017): 1700050. 28798088 10.1183/13993003.00050-2017

[42] N. Guezour , G. Soussi , S. Brosseau , et al., “Grade 3–4 Immune‐Related Adverse Events Induced by Immune Checkpoint Inhibitors in Non‐Small‐Cell Lung Cancer (NSCLC) Patients Are Correlated With Better Outcome: A Real‐Life Observational Study,” Cancers 14 (2022): 14. 10.3390/cancers14163878 PMC9405595 36010872

[43] X. Lin , H. Deng , L. Chen , et al., “Clinical Types of Checkpoint Inhibitor‐Related Pneumonitis in Lung Cancer Patients: A Multicenter Experience,” Translational Lung Cancer Research 10 (2021): 415–429. 33569323 10.21037/tlcr-20-1258 PMC7867788

[44] X. Lin , H. Deng , T. Chu , et al., “Safety and Efficacy of Immunotherapy Rechallenge Following Checkpoint Inhibitor‐Related Pneumonitis in Advanced Lung Cancer Patients: A Retrospective Multi‐Center Cohort Study,” Translational Lung Cancer Research 11 (2022): 2289–2305. 36519018 10.21037/tlcr-22-732 PMC9742619

[45] R. Hamashima , J. Uchino , Y. Morimoto , et al., “Association of Immune Checkpoint Inhibitors With Respiratory Infections: A Review,” Cancer Treatment Reviews 90 (2020): 102109. 33038863 10.1016/j.ctrv.2020.102109

[46] L. Pan , F. Meng , W. Wang , et al., “Nintedanib in an Elderly Non‐Small‐Cell Lung Cancer Patient With Severe Steroid‐Refractory Checkpoint Inhibitor‐Related Pneumonitis: A Case Report and Literature Review,” Frontiers in Immunology 13 (2022): 1072612. 36703957 10.3389/fimmu.2022.1072612 PMC9872202

