# Supplementary material for: Heterogeneity of Checkpoint Inhibitor–Associated Pneumonitis: A Multicenter Study on Inflammatory Subtypes and Clinical Outcomes
Source: Cancer Med. 2025 Jul 16;14(14):e71041. doi: 10.1002/cam4.71041 (PMC12264577; doi:10.1002/cam4.71041)
Supplement: Supplementary file 1 — Figure S1. Clinical features of patients with entire CIP group. (A) Time from first dose of ICIs therapy to the date of each grade CIP developed. (B) Clinical symptoms of CIP. (C) The ICIs of CIP. (D) The survival probability of CIP group and control group. (E) The radiographic classification of CIP. (F) The survival probability of four radiological features. ICIs, immune checkpoint inhibitors; CIP, checkpoint inhibitor pneumonitis. Figure S2. Imaging grades of asymptomatic patients at the onset of CIP. 26.2% patients were in Grade 1, 47.6% in Grade 2, 19.1% in Grade 3 and 7.1% in Grade 4. CIP, checkpoint inhibitor pneumonitis. Figure S3. Radiologic classification of each inflammatory subtype. (A) Neutrophil type; (B) eosinophil type; (C) normal type. Figure S4. Immune checkpoint inhibitors of CIP. (A) Inflammatory subtypes could be induced by different ICIs; (B) Different ICIs could induce different inflammatory subtypes. ICIs, immune checkpoint inhibitors. Figure S5. The survival probability of Inflammatory subtypes. (A) Survival curves of each inflammatory subtype. (B) Comparison of survival curves between neutrophil type and control group. [file CAM4-14-e71041-s001.pptx]

## Slide 1
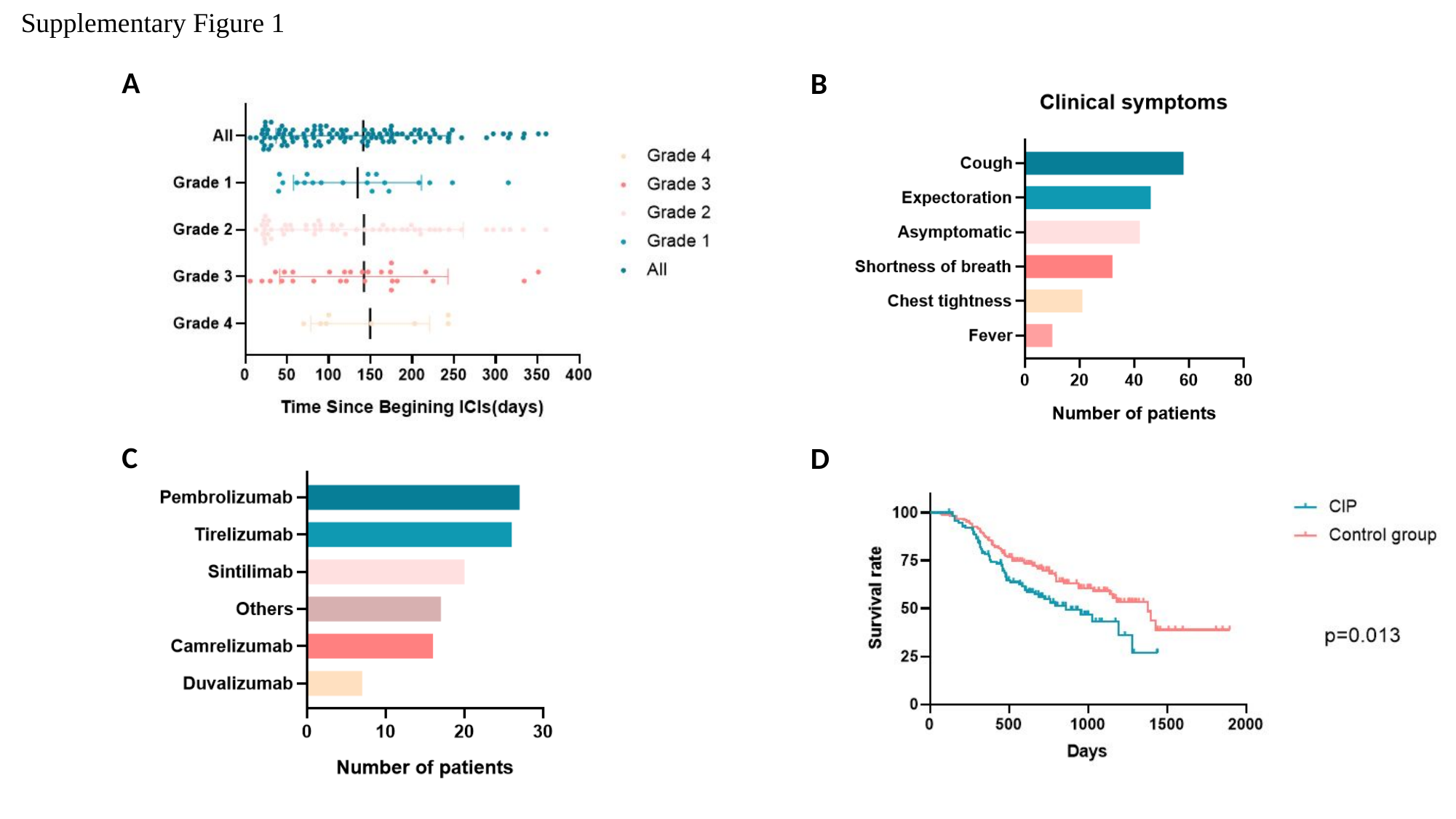

Supplementary Figure 1
A
B
C
D

## Slide 2
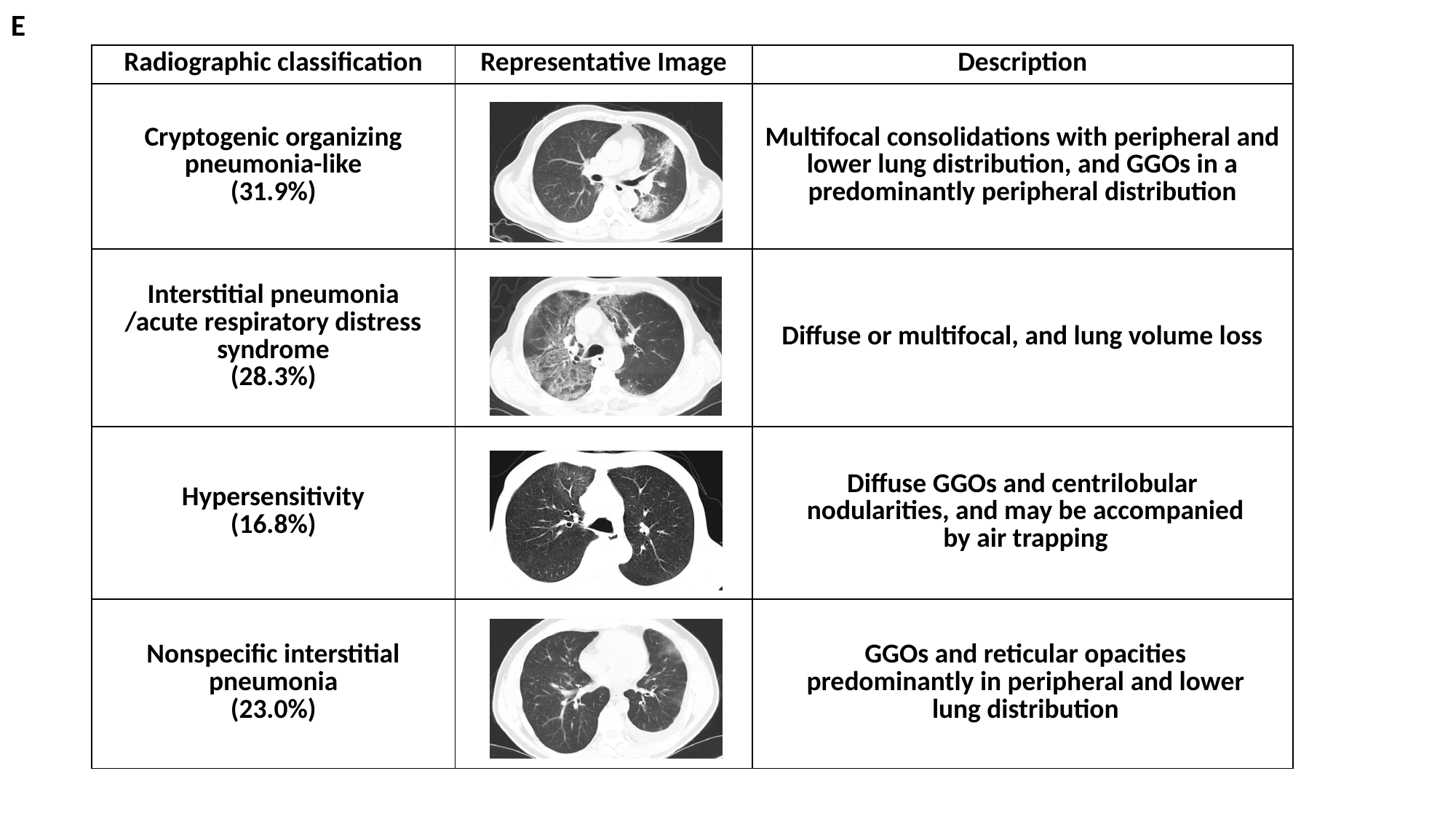

E
| Radiographic classification | Representative Image | Description |
| --- | --- | --- |
| Cryptogenic organizing pneumonia-like (31.9%) | | Multifocal consolidations with peripheral and lower lung distribution, and GGOs in a predominantly peripheral distribution |
| Interstitial pneumonia /acute respiratory distress syndrome (28.3%) | | Diffuse or multifocal, and lung volume loss |
| Hypersensitivity (16.8%) | | Diffuse GGOs and centrilobular nodularities, and may be accompanied by air trapping |
| Nonspecific interstitial pneumonia (23.0%) | | GGOs and reticular opacities predominantly in peripheral and lower lung distribution |

## Slide 3
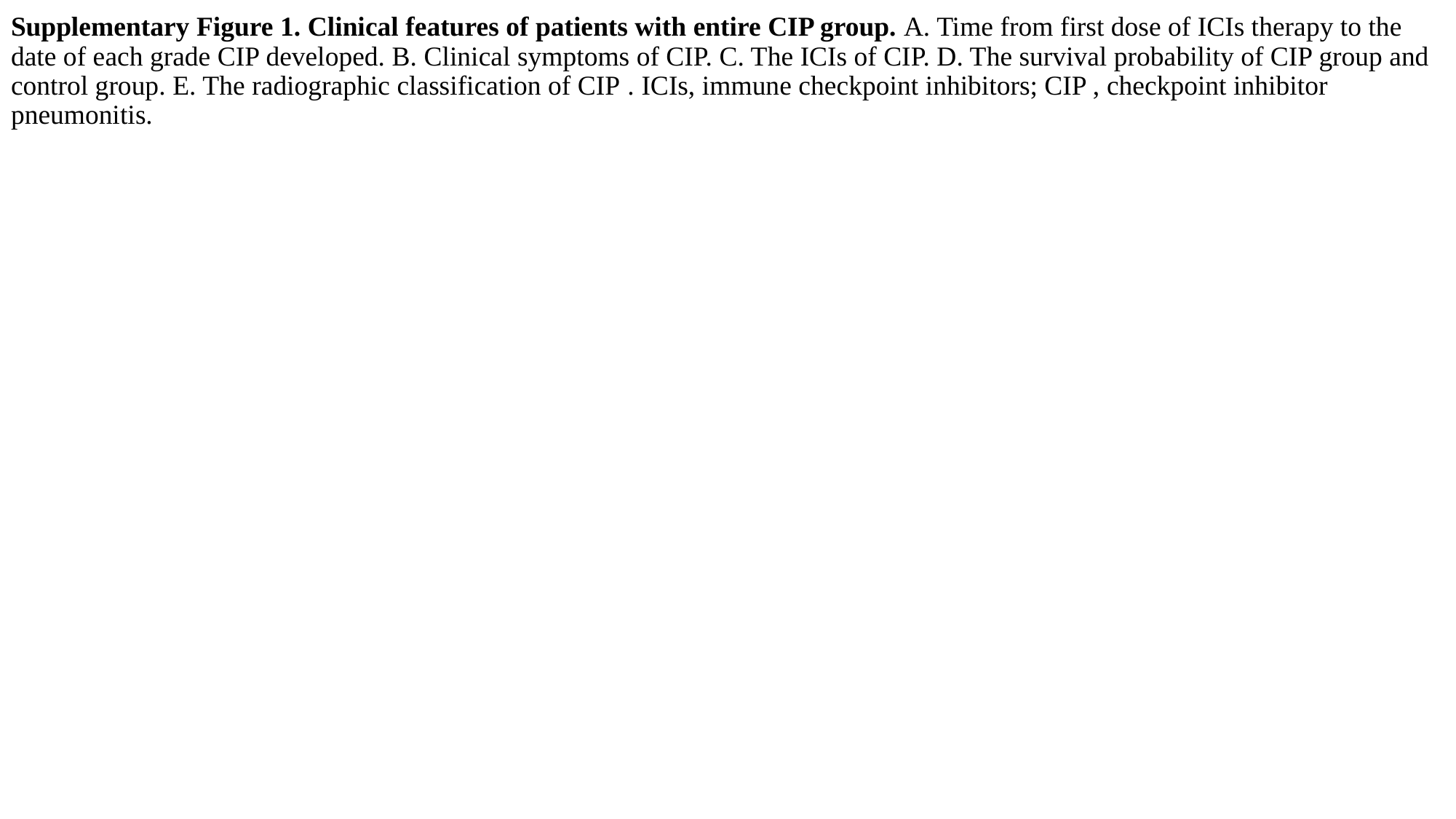

# Supplementary Figure 1. Clinical features of patients with entire CIP group. A. Time from first dose of ICIs therapy to the date of each grade CIP developed. B. Clinical symptoms of CIP. C. The ICIs of CIP. D. The survival probability of CIP group and control group. E. The radiographic classification of CIP . ICIs, immune checkpoint inhibitors; CIP , checkpoint inhibitor pneumonitis.

## Slide 4
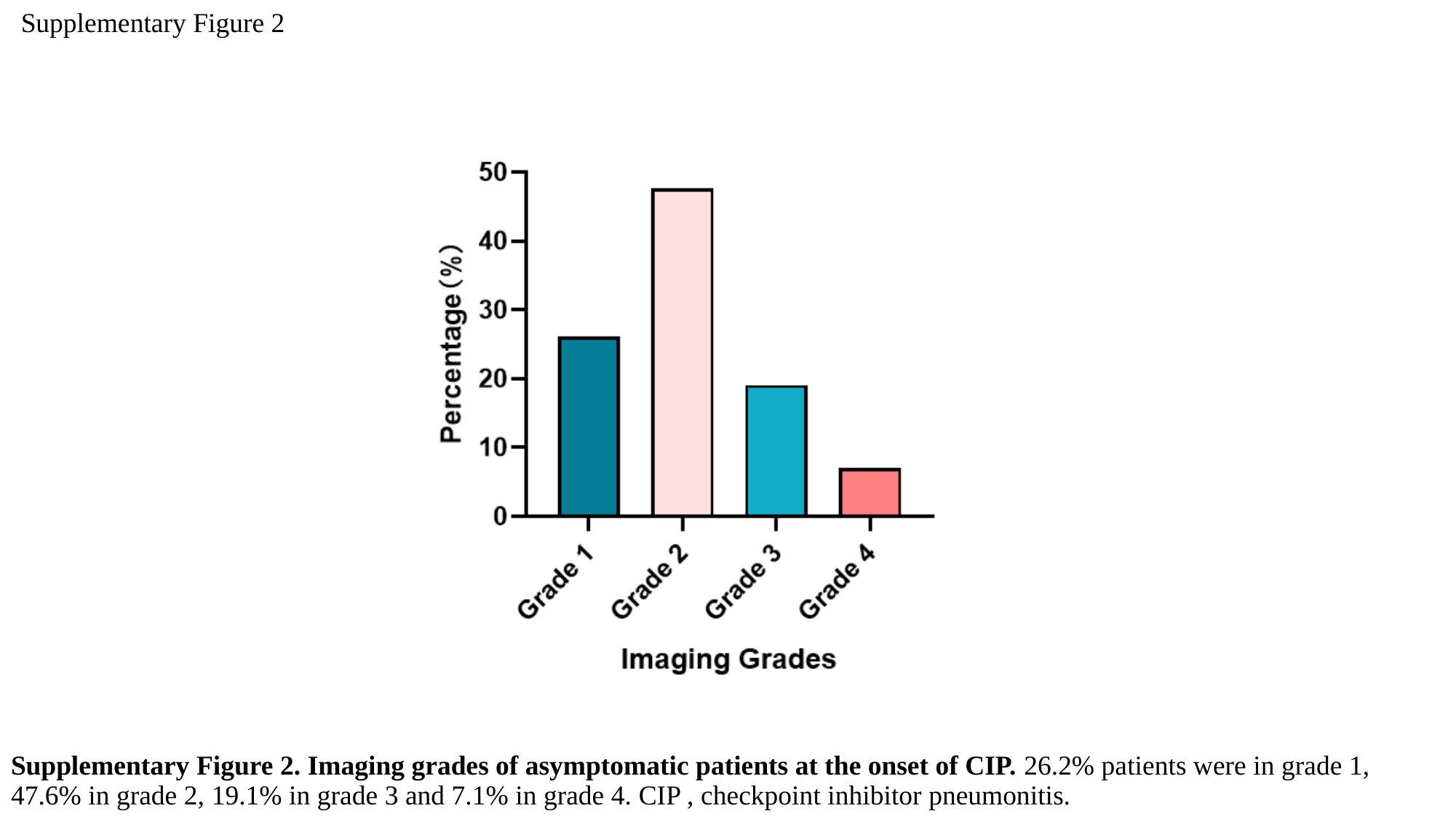

Supplementary Figure 2
# Supplementary Figure 2. Imaging grades of asymptomatic patients at the onset of CIP. 26.2% patients were in grade 1, 47.6% in grade 2, 19.1% in grade 3 and 7.1% in grade 4. CIP , checkpoint inhibitor pneumonitis.

## Slide 5
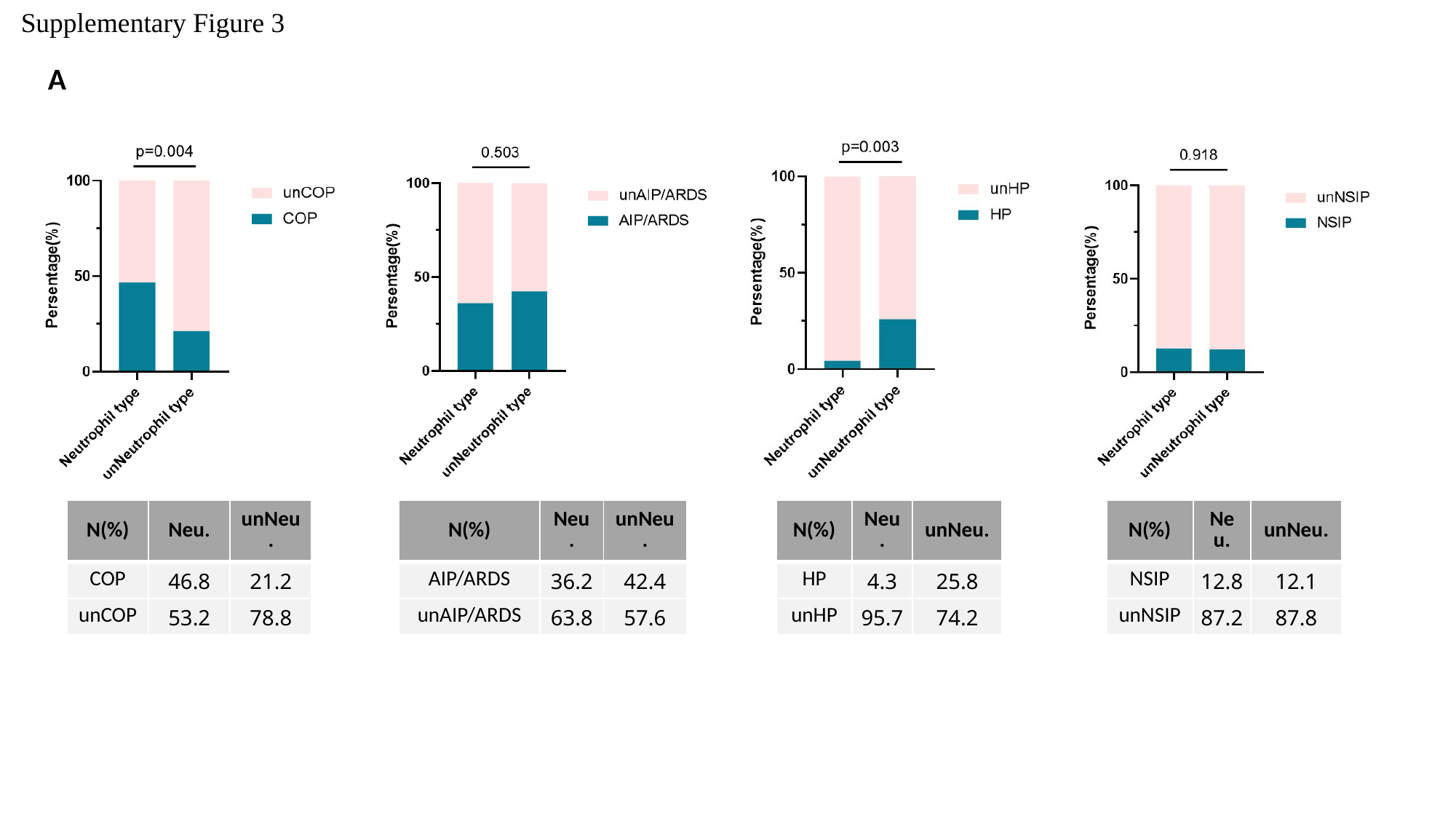

Supplementary Figure 3
A
| N(%) | Neu. | unNeu. |
| --- | --- | --- |
| COP | 46.8 | 21.2 |
| unCOP | 53.2 | 78.8 |
| N(%) | Neu. | unNeu. |
| --- | --- | --- |
| AIP/ARDS | 36.2 | 42.4 |
| unAIP/ARDS | 63.8 | 57.6 |
| N(%) | Neu. | unNeu. |
| --- | --- | --- |
| HP | 4.3 | 25.8 |
| unHP | 95.7 | 74.2 |
| N(%) | Neu. | unNeu. |
| --- | --- | --- |
| NSIP | 12.8 | 12.1 |
| unNSIP | 87.2 | 87.8 |

## Slide 6
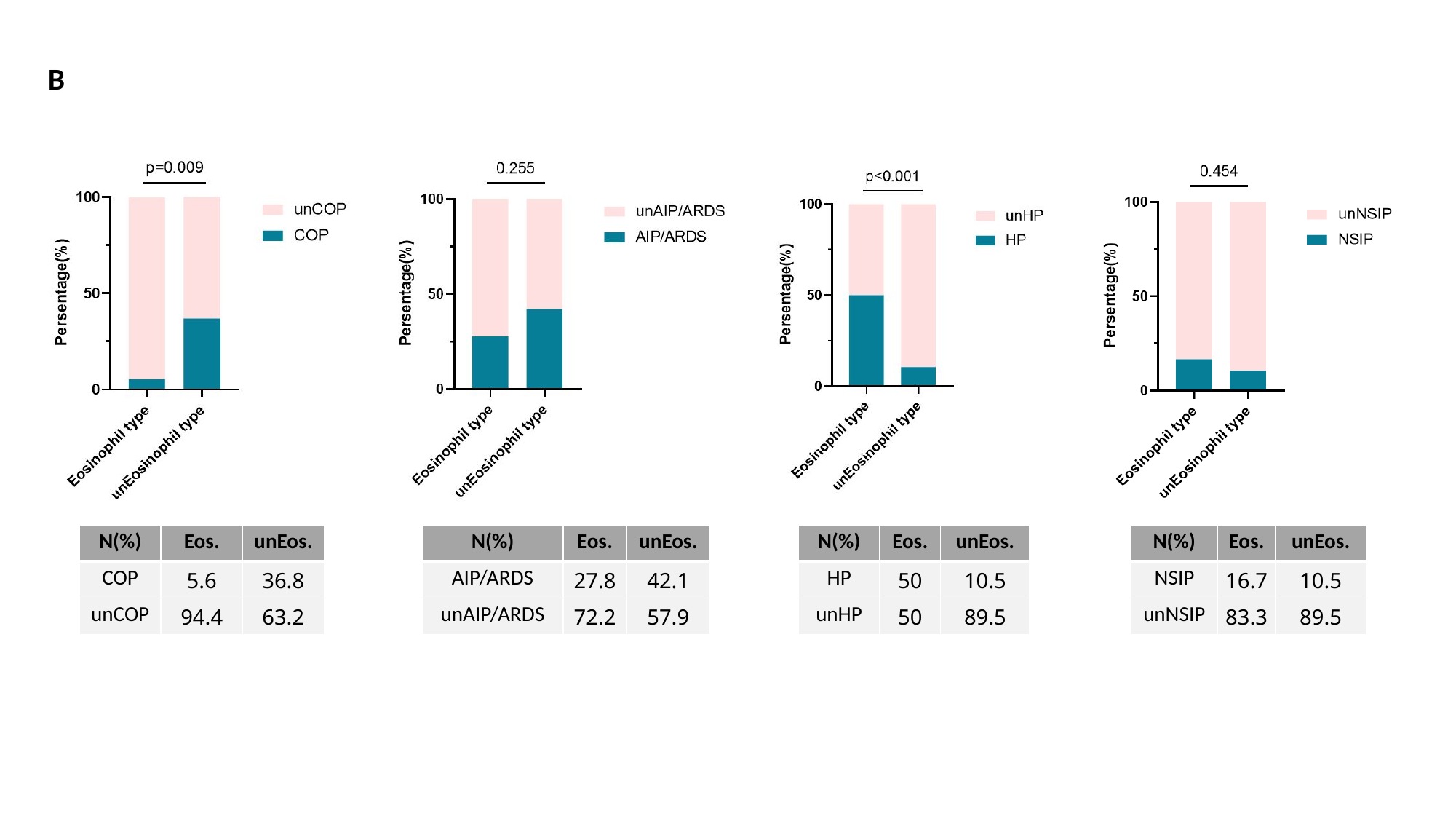

B
| N(%) | Eos. | unEos. |
| --- | --- | --- |
| COP | 5.6 | 36.8 |
| unCOP | 94.4 | 63.2 |
| N(%) | Eos. | unEos. |
| --- | --- | --- |
| AIP/ARDS | 27.8 | 42.1 |
| unAIP/ARDS | 72.2 | 57.9 |
| N(%) | Eos. | unEos. |
| --- | --- | --- |
| HP | 50 | 10.5 |
| unHP | 50 | 89.5 |
| N(%) | Eos. | unEos. |
| --- | --- | --- |
| NSIP | 16.7 | 10.5 |
| unNSIP | 83.3 | 89.5 |

## Slide 7
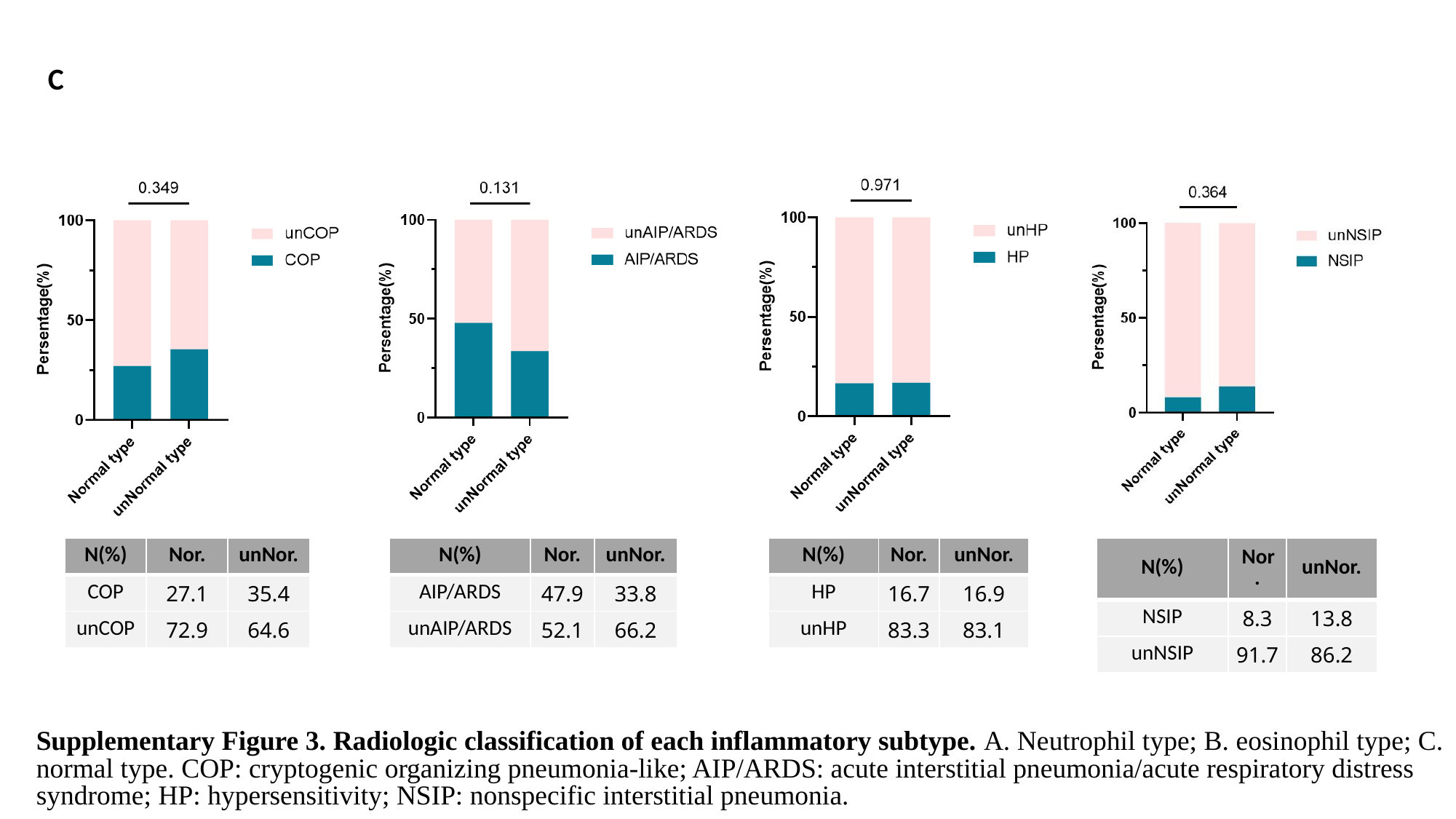

C
| N(%) | Nor. | unNor. |
| --- | --- | --- |
| NSIP | 8.3 | 13.8 |
| unNSIP | 91.7 | 86.2 |
| N(%) | Nor. | unNor. |
| --- | --- | --- |
| COP | 27.1 | 35.4 |
| unCOP | 72.9 | 64.6 |
| N(%) | Nor. | unNor. |
| --- | --- | --- |
| AIP/ARDS | 47.9 | 33.8 |
| unAIP/ARDS | 52.1 | 66.2 |
| N(%) | Nor. | unNor. |
| --- | --- | --- |
| HP | 16.7 | 16.9 |
| unHP | 83.3 | 83.1 |
Supplementary Figure 3. Radiologic classification of each inflammatory subtype. A. Neutrophil type; B. eosinophil type; C. normal type. COP: cryptogenic organizing pneumonia-like; AIP/ARDS: acute interstitial pneumonia/acute respiratory distress syndrome; HP: hypersensitivity; NSIP: nonspecific interstitial pneumonia.

## Slide 8
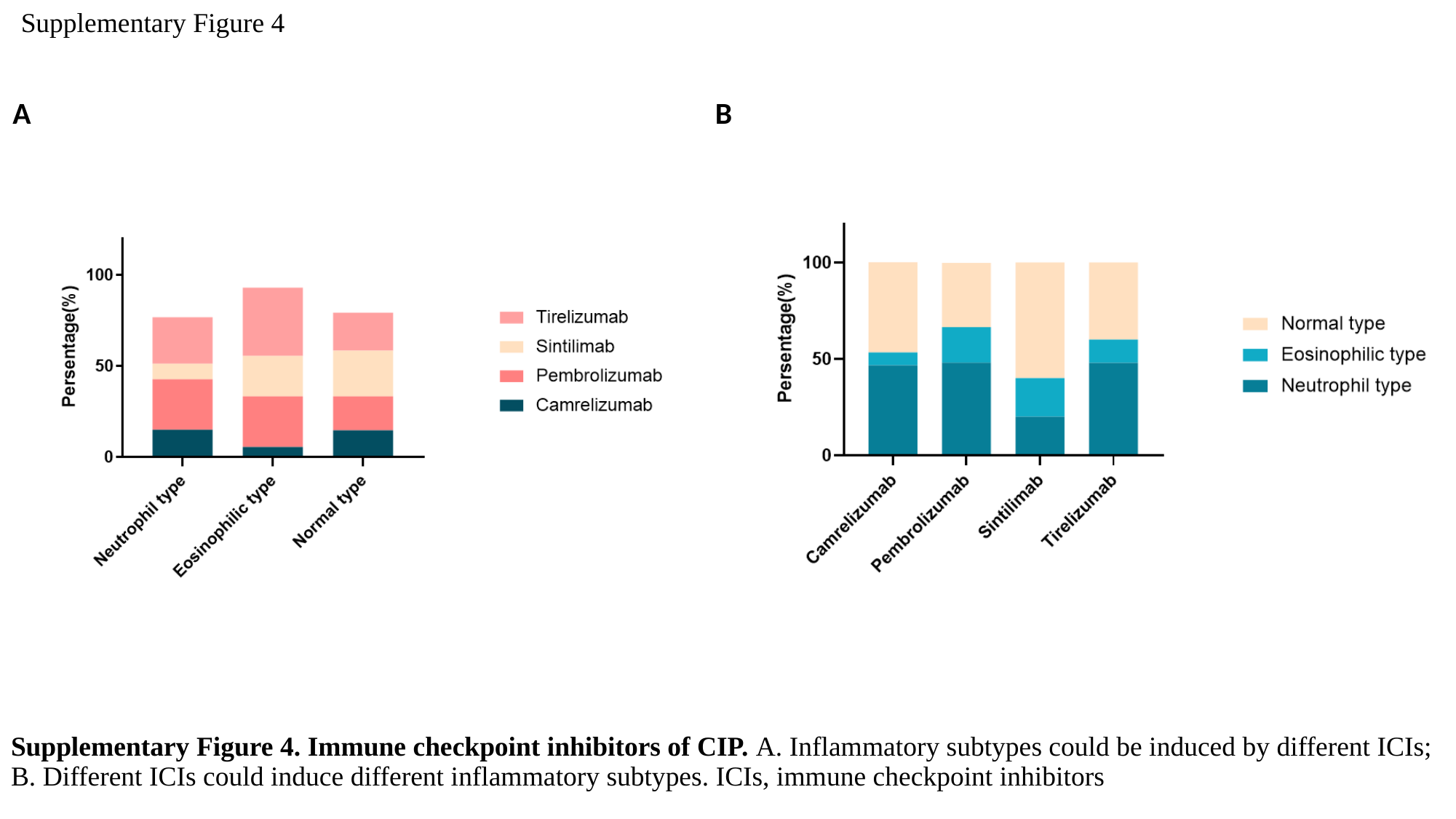

Supplementary Figure 4
A
B
Supplementary Figure 4. Immune checkpoint inhibitors of CIP. A. Inflammatory subtypes could be induced by different ICIs; B. Different ICIs could induce different inflammatory subtypes. ICIs, immune checkpoint inhibitors

## Slide 9
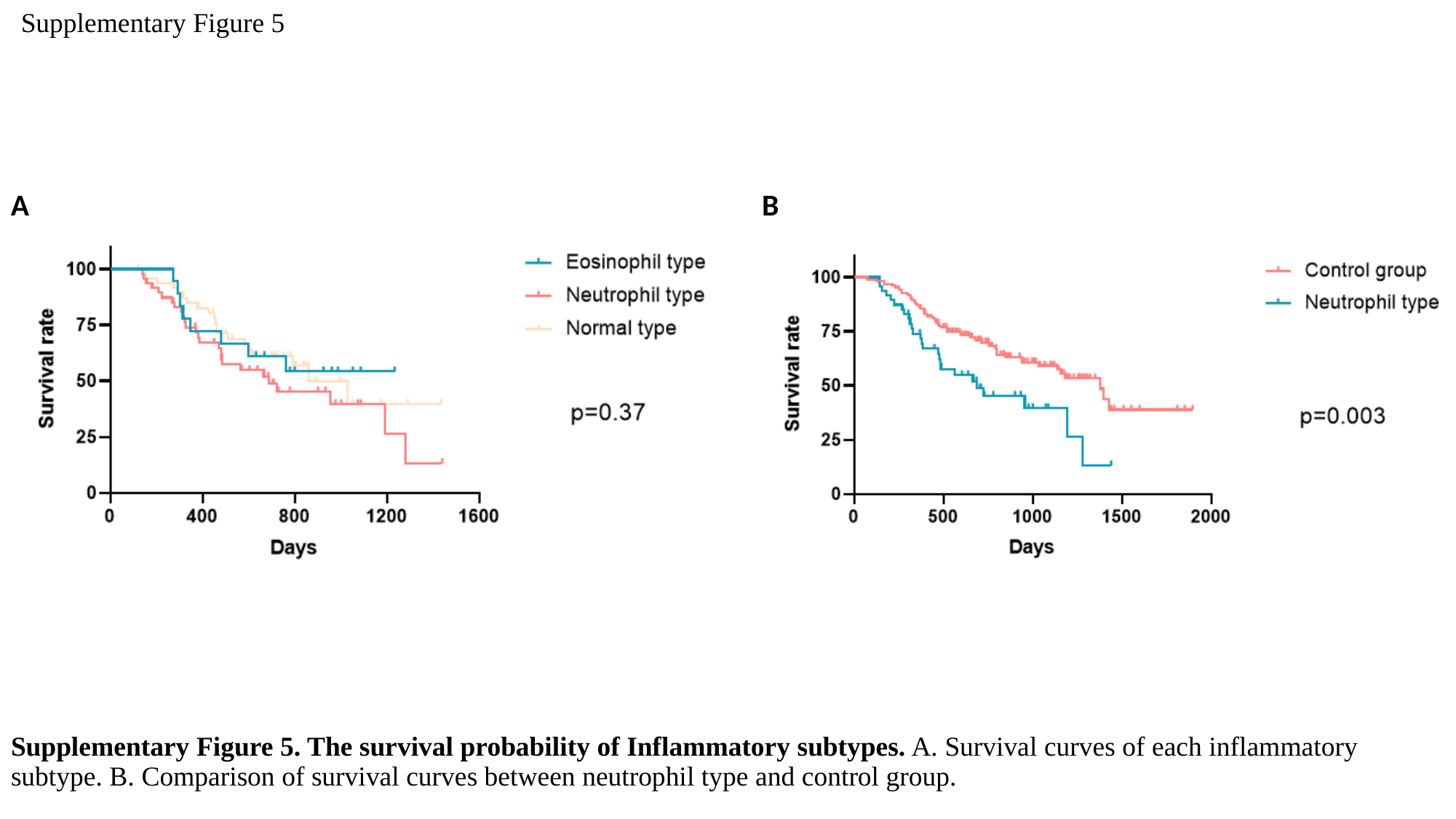

Supplementary Figure 5
A
B
Supplementary Figure 5. The survival probability of Inflammatory subtypes. A. Survival curves of each inflammatory subtype. B. Comparison of survival curves between neutrophil type and control group.
