# Supplementary material for: Heterogeneity of Checkpoint Inhibitor–Associated Pneumonitis: A Multicenter Study on Inflammatory Subtypes and Clinical Outcomes
Source: Cancer Med. 2025 Jul 16;14(14):e71041. doi: 10.1002/cam4.71041 (PMC12264577; doi:10.1002/cam4.71041)
Supplement: Supplementary file 2 — Table S1. Baseline characteristics of the study population. [file CAM4-14-e71041-s002.docx]

**Supplementary Table 1. Baseline characteristics of the study population.**

|  | | | |
| --- | --- | --- | --- |
| **Variables (n, (%))** | **CIP group (n=113)** | **Control group (n=152)** | **P-value** |
| **Gender** |  |  | 0.884 |
| Male | 101 (89.4) | 135 (88.8) |  |
| Female | 12 (10.6) | 17 (11.2) |  |
| **Age at CIP diagnosis/included** |  |  | 0.922 |
| <65 | 64 (56.6) | 87 (57.2) |  |
| ≥65 | 49 (43.4) | 65 (42.8) |  |
| **Smoking habits** |  |  | **0.017** |
| Current | 26 (23.0) | 42 (27.6) |  |
| Former | 68 (60.2) ^a^ | 66 (43.4) ^b^ |  |
| Never | 19 (16.8) ^a^ | 44 (28.9) ^b^ |  |
| **History of lung disease** |  |  | **<0.001** |
| Chronic pulmonary inflammation | 36 (31.9) ^a^ | 14 (9.2) ^b^ |  |
| Emphysema | 28 (24.8) | 40 (26.3) |  |
| Lung nodules | 22 (19.5) ^a^ | 4 (2.6) ^b^ |  |
| Others* | 4 (3.5) | 11 (7.2) |  |
| None | 23 (20.4) ^a^ | 85 (55.9) ^b^ |  |
| **Primary cancer type** |  |  | 0.600 |
| Lung cancer |  |  |  |
| Adenocarcinoma | 40 (35.4) | 50 (32.9) |  |
| Squamous | 32 (28.3) | 42 (27.6) |  |
| Small cell carcinoma | 23 (20.4) | 26 (17.1) |  |
| Others# | 18 (15.9) | 34 (22.4) |  |
| **PD-L1 expression status** |  |  | 0.792 |
| <1% | 21 (18.6) | 30 (19.7) |  |
| 1-49% | 29 (25.7) | 36 (23.7) |  |
| ≥50% | 14 (12.4) | 25 (16.4) |  |
| Not examined | 49 (43.4) | 61 (40.1) |  |
| **ICIs treatment line** |  |  | 0.521 |
| 1 | 80 (70.8) | 113(74.3) |  |
| ≥2 | 33(29.2) | 39(25.7) |  |
| **Therapeutic regimen** |  |  | 0.351 |
| ICIs combination therapy^ | 93 (82.3) | 118 (77.6) |  |
| ICIs monotherapy | 20 (17.7) | 34 (22.4) |  |
| PD-L: programmed cell death ligand; ICIs, immune checkpoint inhibitor. | | | |
| *Others included atelectasis and pulmonary embolism. | | | |
| #Others included gastric cancer, esophageal cancer and nasopharyngeal cancer, etc.  ^Combination therapy including cisplatin+pemetrexed or carboplatin+paclitaxel,etc.  a, b: a statistical difference between the two groups. | | | |
